# ﻿Distribution patterns of rDNA loci in the *Schedonorus*-*Lolium* complex (Poaceae)

**DOI:** 10.3897/compcytogen.v16.i1.79056

**Published:** 2022-03-24

**Authors:** Helal Ahmad Ansari, Nicholas Ellison, Alan Vincent Stewart, Warren Mervyn Williams

**Affiliations:** 1 AgResearch Ltd, Grasslands Research Centre, Palmerston North 4412, New Zealand Grasslands Research Centre Palmerston North New Zealand; 2 PGG Wrightson Seeds, Kimihia Research Centre, 1375 Springs Road, RD4, Lincoln 7674, New Zealand Kimihia Research Centre Lincoln New Zealand

**Keywords:** *Festuca*, FISH, karyotype evolution, *Lolium*, rDNA locus evolution, species diversification

## Abstract

The *Schedonorus*-*Lolium* complex of the subtribe Loliinae (Poaceae) includes several economically important forage and turf grasses. This complex encompasses *Lolium* Linnaeus, 1753, Festuca Linnaeus, 1753 subgenus Schedonorus (P. Beauvois, 1824) Petermann, 1849 and *Micropyropsis* Romero Zarco et Cabezudo, 1983. New FISH results of 5S and 18S–26S rDNA sequences are presented for three species and the results are interpreted in a review of distribution patterns of 5S and 18S–26S rDNA sequences among other species in the complex. *Micropyropsistuberosa* Romero Zarco et Cabezudo, 1983 (2*n* = 2*x* = 14) displayed a distribution pattern of rDNA sequences identical to that of *F.pratensis* Hudson, 1762, supporting a close phylogenetic relationship at the bottom of the phylogenetic tree. “*Loliummultiflorum*” Lamarck, 1779 accessions sourced from Morocco showed a different pattern from European *L.multiflorum* and could be a unique and previously uncharacterised taxon. North African *Festucasimensis* Hochstetter ex A. Richard, 1851 had a marker pattern consistent with allotetraploidy and uniparental loss of one 18S–26S rDNA locus. This allotetraploid has previously been suggested to have originated from a hybrid with *Festucaglaucescens* (Festucaarundinaceavar.glaucescens Boissier, 1844). However, the distribution patterns of the two rDNA sequences in this allotetraploid do not align with *F.glaucescens*, suggesting that its origin from this species is unlikely. Furthermore, comparisons with other higher alloploids in the complex indicate that *F.simensis* was a potential donor of two sub-genomes of allohexaploid *Festucagigantea* (Linnaeus) Villars, 1787. In the overall complex, the proximal locations of both rDNA markers were conserved among the diploid species. Two types of synteny of the two markers could, to a considerable extent, distinguish allo- and autogamous *Lolium* species. The ancestral parentage of the three *Festuca* allotetraploids has not yet been determined, but all three appear to have been sub-genome donors to the higher allopolypoids of sub-genus *Schedonorus*. Terminal locations of both the markers were absent from the diploids but were very frequently observed in the polyploids.

## ﻿Introduction

Ryegrasses of the genus *Lolium* Linnaeus, 1753 with ten diploid species and fescues of the genus Festuca Linnaeus, 1753 subgenus Schedonorus (P. Beauvois, 1824) Petermann, 1849 are closely related and, together with *Micropyropsis* Romero Zarco et Cabezudo, 1983, form the “*Schedonorus*-*Lolium* complex”, belonging to the family Poaceae Barnhart, 1895, subtribe Loliinae Dumortier, 1829 (Inda et al. 2013; Cheng et al. 2016). Several of these *Lolium* and *Festuca* species, which are native to temperate regions of Europe, Asia and Africa, are widely used for forage and turf purposes in all major temperate regions of the planet. *Micropyropsistuberosa* Romero Zarco et Cabezudo, 1983 (Romero Zarco and Cabezudo 1983) is the sole species of the genus and is diploid (Romero Zarco 1988).

Since the last major taxonomic revision of the genus *Lolium* by Terrell (1968), new species have been discovered and named, notably *Loliumsaxatile* H. Scholz et S. Scholz, 2005 (Scholz and Scholz 2005) and *Loliumedwardii* H. Scholz, Stierstorfer et van Gaisberg, 2000 (Scholz et al. 2000). Although *Festuca* has over 500 diploid to dodecaploid species, subgenus Schedonorus is limited to approximately 20 species, most from Europe, W Asia or N Africa. However, the broad-leaved *Festuca* species from highland tropical Africa, including *Festucasimensis* Hochstetter ex A. Richard, 1851 have also been shown to be part of the *Schedonorus*-*Lolium* complex (Namaganda et al. 2006; Inda et al. 2014; Minaya et al. 2015).

Several molecular genetic analyses involving DNA markers have been successfully carried out for the phylogenetic reconstruction of subtribe Loliinae. It has been shown that the *Schedonorus*-*Lolium* complex represents a monophyletic group, with *Lolium* clearly differentiated from *Festuca* (Charmet et al. 1997; Gaut et al. 2000; Catalán et al. 2004; Namaganda et al. 2006; Hand et al. 2010; Inda et al. 2014; Minaya et al. 2015; Cheng et al. 2016). Fertile hybrids formed between *Lolium* and *Festuca* species show chromosome pairing and recombination but the chromosomes can be distinguished using genomic *in situ* hybridization (Humphreys et al. 1995).

Karyological differences featuring chromosome number, structure and morphology have long been used to infer the systematic status and the evolutionary history of species divergence. However, in some groups of species conventionally stained chromosome preparations do not clearly delineate structural differences among chromosomes or species karyotypes. Molecular cytogenetic mapping of specific DNA sequences through fluorescence *in situ* hybridization (FISH) can overcome such problems, and provide enhanced pictures of chromosome architecture, leading to clear karyotype and genome discrimination (Albert et al. 2010; Chester et al. 2010; Xiong and Pires 2011). Two different families of multicopy and highly conserved ribosomal RNA genes (rDNA), one coding for 5S and the other for 35S rRNA arrays are universally present in plants. Tandemly repeated blocks of these genes are located independently at particular chromosomal sites and provide species-specific markers (Roa and Guerra 2015). Each 35S rDNA unit carries 18S, 5.8S and 26S RNA genes along with two internal transcribed spacers (ITSs) and tandemly repeated blocks of these units form the nucleolar organizer regions (NORs) or secondary constrictions on chromosomes. FISH mapping of 5S and 35S rDNA sequences is widely used to compare the chromosomal structural changes of related species and to infer the karyoevolutionary variations that accompany species diversification (Fukushima et al. 2011; Lan and Albert 2011; Roa and Guerra 2012, 2015; Jang et al. 2013).

Species of the *Schedonorus*-*Lolium* complex all share *x* = 7 as the base chromosome number and all have very similar biarmed chromosome morphologies and symmetrical karyotypes. Therefore, conventional karyological information is of little value for evaluating evolutionary changes (Malik and Thomas 1966; Namaganda et al. 2006; Kopecký et al. 2010). Molecular cytogenetic mapping of 5S and 35S rDNA has detected variations in the distributional patterns of the two rDNA markers among diploids and polyploids in this complex (Thomas et al. 1996, 1997; Ksia¸zczyk et al. 2010; Inda and Wolny 2013; Ansari et al. 2016; Ezquerro-López et al. 2017; Shafiee et al. 2020). Based on their report, Ezquerro-López et al. (2017) made a preliminary attempt to decipher the evolutionary relationships among *Festuca* species belonging to this complex.

In this study, we have mapped the chromosomal dispositions of 5S and 18S rDNA loci in five taxa, three of which were previously unmapped, and have discussed the evolutionary implications of the new results. Following this we have drawn together all the available information from disparate sources and have framed a more complete picture of rDNA chromosome patterns within the whole of this economically important complex. This is the first time such information has been integrated across numerous studies.

## ﻿Methods

### ﻿Plant materials and chromosome preparations

Seeds from five populations (Table 1) belonging to the *Schedonorus*-*Lolium* complex were accessed from the Margot Forde Forage Germplasm Centre at AgResearch Grasslands, Palmerston North and PGG Wrightson Seeds, Christchurch, New Zealand. *Loliummultiflorum* Lamarck, 1779 of Moroccan origin was designated MRCN to distinguish it from *L.multiflorum* material of European origin. Seeds were germinated and grown in a glasshouse. Somatic chromosome preparations were obtained from the meristematic tissue of actively growing root tips according to the flame-drying technique described earlier (Ansari et al. 1999, 2016). Good quality cytological preparations were selected after screening using phase contrast optics.

**Table 1.** List of *Schedonorus*-*Lolium* complex taxa used in this study.

Taxon	Identity and source of seed
*Festucasimensis* Hochstetter ex A. Richard, 1851	BL 2043, Margot Forde Forage Germplasm Centre
*Loliumperenne* Linnaeus, 1753	Cv Impact, Margot Forde Forage Germplasm Centre
*Loliummultiflorum* Lamarck, 1779	B 3380, Margot Forde Forage Germplasm Centre
*Loliummultiflorum* MRCN	Cv. Barberia, PGG Wrightson Seeds
*Micropyropsistuberosa* Romero Zarco et Cabezudo, 1983	BZ 8319, Margot Forde Forage Germplasm Centre

### ﻿Fluorescence *in situ* hybridization (FISH)

The DNA probes used for FISH were pTr18S (GenBank accession number AF071069), a 1.8 kb fragment from *Trifoliumrepens* Linnaeus, 1753 containing almost the entire 18S rDNA sequence representing the 35S rDNA and pTr5S (GenBank accession number AF072692), a 596 bp DNA fragment encoding the *T.repens* 5S rRNA. 35S and 5S rDNA probes were directly labelled with fluorochromes Fluor-X-dCTP and Cy-3-dCTP (GE Healthcare, NZ), respectively by nick translation according to manufacturer’s specifications. Double target FISH using the above DNA probes, post-hybridisation washing and counterstaining of somatic chromosomes with DAPI were carried out as described earlier (Ansari et al. 1999). Chromosome preparations were mounted in Vectashield (Vector Laboratories). Fluorescence images were acquired using a Zeiss monochrome MRm CCD camera on a Nikon epifluorescence microscope Microphot-SA and were processed with an ISIS FISH Imaging System (MetaSystems, Germany). At least five good quality early to late metaphase cells from each plant were used for analysing hybridization signals.

## ﻿Results

**Figure 1.**
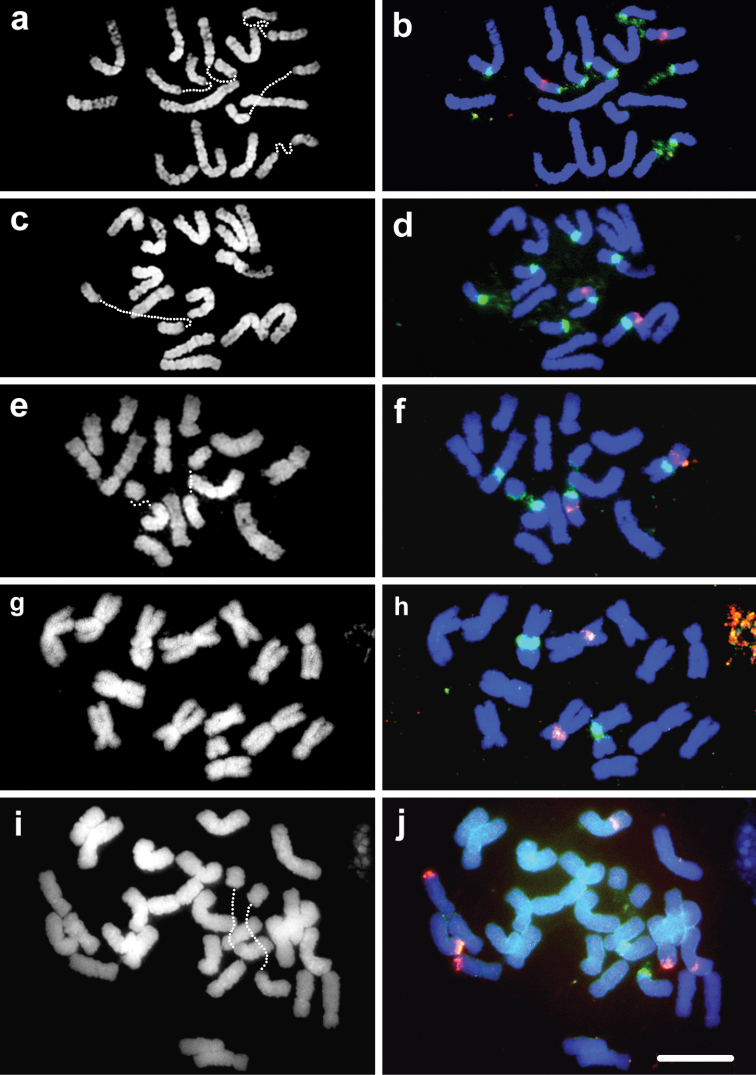
DAPI stained (grey scale) metaphase cells in the left column and the same cells in the right column displaying FISH mapping of 5S (red signals) and 35S rDNA sequences (green signals) in **a, b***L.perenne***c, d***L.multiflorum*, European origin **e, f***L.multiflorum* MRCN Moroccan origin **g, h***M.tuberosa***i, j***F.simensis*. Dotted lines in **a, c, e, g**, and **i** denote decondensed 35S rDNA chromatin.

Results of double colour FISH mapping using 35S and 5S rDNA sequences as probes on pro-metaphase or metaphase chromosomes of *Loliumperenne* Linnaeus, 1753 (2*n* = 2*x* = 14) are given in Fig. 1. Six 35S rDNA signals representing three loci were located proximally on three pairs of chromosomes (Fig. 1a, b). One locus was on the short arm of one chromosome pair, and the other two displayed hybridization on the long arms of two pairs of chromosomes. One of the chromosome pairs with 35S on the long arm displayed co-localization of the single 5S rDNA locus proximally on the short arm. The chromatin housing 35S rDNA regions, representing GC-rich nucleolus organizer regions (NORs) or secondary constrictions, were frequently decondensed and sometimes stretched in our flame-dried somatic chromosome preparations. These loci are positioned pericentromerically, and the cloudy decondensed and stretched 35S rDNAFISH signals could be observed joining the two condensed parts of NOR-bearing chromosomes (Fig. 1a, b). *L.multiflorum* (2*n* = 2*x* = 14) of north European/Mediterranean origin produced rDNAFISH signals identical to the pattern observed for *L.perenne* (Fig. 1c, d).

In contrast to *L.perenne* and *L.multiflorum* of north European origin, *L.multiflorum* (2*n* = 2*x* = 14) of Moroccan origin displayed only two pairs of NORs (Fig. 1e, f), each pair located proximally on the long arm. One of these NOR-bearing chromosome pairs co-localised 5S sequences proximally on the short arm.

*Micropyropsistuberosa*, 2*n* = 2*x* = 14, with a symmetrical karyotype, displayed one 5S and one 35S rDNA locus, each on separate chromosome pairs, and located proximally on the short arms (Fig. 1g, h). Co-localization of the two rDNA sequences on the same chromosome was not observed in *M.tuberosa*.

*Festucasimensis*, 2*n* = 4*x* = 28, displayed all biarmed chromosomes and a symmetrical karyotype. The eight FISH signals were distributed on separate chromosomes (Fig. 1i, j). One of the three pairs of 5S rDNA signals hybridized interstitially on the short arms. Each of the remaining two pairs of 5S signals were located distally in terminal regions, one in the short arm and the other in the long arm of two pairs of chromosomes. The only pair of 35S signals was located proximally on the short arms of a chromosome pair. Again, *F.simensis* did not show co-localization of the two rDNA sequences.

## ﻿Discussion

We have mapped the diversity in the chromosomal locations of the two rDNA sequences for five taxa of the *Schedonorus*-*Lolium* complex. Three of these, *M.tuberosa*, *L.multiflorum* MRCN and *F.simensis*, were previously unmapped. The results for *L.perenne* and N European *L.multiflorum* agree with previous studies (Thomas et al. 1996; Ansari et al. 2016). The new results are discussed first and then rDNA chromosomal patterns across the complex are reviewed.

*Micropyropsistuberosa* exhibited single 5S and 35S rDNA loci positioned proximally on separate chromosomes as was also the case for *F.pratensis* (Thomas et al. 1997). In phylogenetic reconstructions within the *Schedonorus*-*Lolium* complex based on ITS and plastid DNA sequences, the divergence of *M.tuberosa* preceded the basal split between the diploid lineages of *Festuca* and *Lolium* (Torrecilla and Catalán 2002; Catalán et al. 2004; Inda et al. 2008, 2014; Šmarda et al. 2008). The similar arrangement of single 5S and 35S rDNA loci in *M.tuberosa* and *F.pratensis* is consistent with the interpretation that this was the ancestral diploid *Schedonorus* arrangement before the *Lolium* split.

The “*L.multiflorum*” of Moroccan origin is typical of the main *Lolium* lineage in having more than one 35S rDNA locus. One of these 35S loci has a syntenic 5S locus on the opposite chromosome arm, in common with *L.perenne* and *L.multiflorum* of Eurasian origin. However, compared with Eurasian *L.multiflorum* the Moroccan taxon has one fewer 35S locus. The Moroccan “*L.multiflorum*” could be a new and unique N African taxon that has chromosomal affinities with the allogamous Eurasian *Lolium* species.

**Figure 2.**
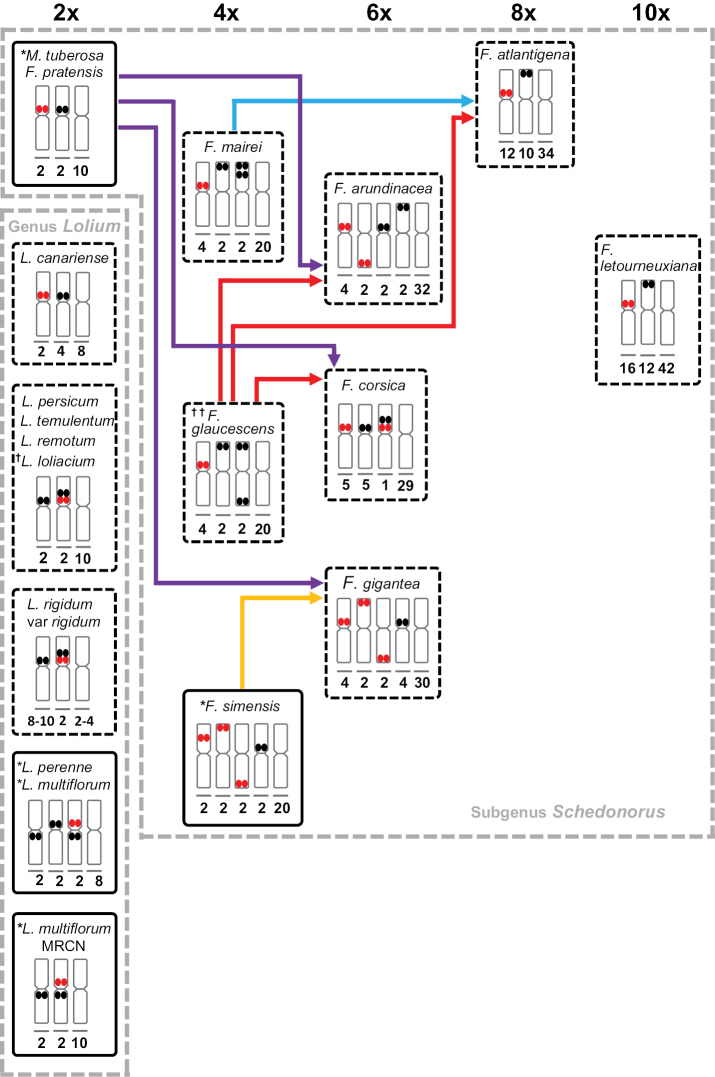
Schematic representation of the putative evolutionary lineages for chromosomes carrying 5S and 35S rDNA loci in the *Schedonorus-Lolium* complex. The numbers of marker and non-marker chromosomes are given inside the boxes. Red and black double circles represent 5S and 35S rDNA loci, respectively. *species in solid boxes were investigated during the present study; **^†^**synonym for L.rigidumvar.rottbollioides; **^††^**synonym for F.arundinaceasubsp.fenas (Lagasca y Segura) Bornmüller, 1928 (Ezquerro-López et al. 2017).

A previous cytological analysis of the tropical African broad-leaved fescue, *F.simensis*, showed it to be tetraploid (2*n* = 4*x* = 28) and AFLP fingerprinting revealed a close phylogenetic relationship with European broad-leaved fescues, especially with hexaploid *F.gigantea*, (Namaganda et al. 2006). Nuclear and plastid DNA sequence studies also placed *F.simensis* in the *Schedonorus*-*Lolium* complex, close to *Lolium* (Inda et al. 2014). In this first molecular cytogenetics analysis of *F.simensis*, we have confirmed the tetraploidy, revealed a symmetrical biarmed karyotype and a distributional pattern of the two rDNA sequences consistent with allopolyploidy (Figs 1 and 2). In addition to two terminal 5S loci, on separate chromosomes, an interstitial 5S locus was observed on the short arm of a separate chromosome, a new location for this group of fescues. None of these 5S positions was consistent with the suggested close relationship with *Lolium*. On the other hand, the 35S rDNA locus was positioned proximally and could represent a link with a common ancestor to *Lolium*. Only one 35S locus was encountered in this allotetraploid, indicating uniparental loss during diploidisation. There are numerous examples of uniparental loss of 35S loci occurring in other allopolyploids (Ansari et al. 1999; Kotseruba et al. 2003, 2010; Williams et al. 2012; Kolano et al. 2016).

Based on a low-copy nuclear gene analysis, Minaya et al. (2015) suggested a Mediterranean origin of Afromontane *F.simensis* through hybridization between a diploid *F.glaucescens* and a *Lolium*-like diploid species. However, none of the distribution patterns of the two rDNA sequences in this allotetraploid align with *F.glaucescens* (Festucaarundinaceavar.glaucescens Boissier, 1844). Instead, the distribution patterns are consistent with the possible involvement of *F.simensis* in the formation of 6x *F.gigantea* (Linnaeus) Villars, 1787. *Festucapratensis* Hudson, 1762 is a putative diploid sub-genome donor of allohexaploid *F.gigantea* (Hand et al. 2010), but the sources of the other subgenomes remain unknown. We have noted a close similarity between the 5S and 35S patterns of allotetraploid *F.simensis* (present results) and *F.gigantea* (Thomas et al. 1997, Fig. 2). These species also show a close phylogenetic proximity based on DNA sequences (Namaganda et al. 2006; Inda et al. 2014). Hence, we infer that allotetraploid *F.simensis* could be a potential donor of the remaining two sub-genomes of allohexaploid *F.gigantea* (Fig. 2).

### ﻿rDNA locus patterns across the diploid *Schedonorus*-*Lolium* taxa

All *Lolium* species, along with *M.tuberosa* and *F.pratensis* are natural diploids. The *Lolium* species, are evolutionarily more recent than the *Festuca* species based on DNA sequence phylogenies (Gaut et al. 2000; Catalan et al. 2004; Inda et al. 2014). All *Lolium* taxa studied so far, comprising eight of the ten extant species, displayed exclusively proximal chromosomal locations of both 5S and 35S rDNA sequences (Fig. 2). After the divergence from *Festuca*, the *Lolium* lineage invariably conserved the proximal locations of both the rDNA loci, but changes in the numbers and syntenic status of these loci apparently occurred later. The proximal localization of 5S rDNA in these diploids matches well with the general distribution pattern of this locus among angiosperms but contrasts with most Poaceae (Roa and Guerra 2015). The proximal mapping of 35S loci contrasts with more terminal localizations in the majority of angiosperms, including Poaceae (Roa and Guerra 2012; Garcia et al. 2017).

A single 5S rDNA locus (two FISH signals per cell) consistently occurred in all *Lolium* species. The number of 35S loci displayed has previously been noted as a distinguishing feature between *F.pratensis* (one locus) and *Lolium* species (more than one locus) (Thomas et al. 1996; Inda and Wolny 2013). All the *Lolium* taxa displayed increases in the number of 35S loci ranging from 2 to 5 (Fig. 2). Accordingly, there are two loci in *L.multiflorum* (Moroccan origin), *L.persicum* Boissier et Hohenacker, 1854, *L.temulentum* Linnaeus, 1753, *L.remotum* Schrank, 1789, L.rigidumvar.rottbollioides Heldreich ex Boissier, 1884 and *L.canariense* Steudel, 1855, three in *L.perenne* and *L.multiflorum* (European origin) to four or five in L.rigidumvar.rigidum Gaudin, 1811. These results were consistent with those of angiosperms in general, where numbers of 5S sites vary considerably less than 35S sites (Lan and Albert 2011; Garcia et al. 2017).

The two types of rDNA loci can be located on the same chromosome (syntenic) or on separate chromosomes (non-syntenic) (Morales et al. 2012; Barros e Silva et al. 2013; Olanj et al. 2015). The Macaronesian *Lolium* species, *L.canariense*, has no synteny of 5S and 35S loci (Inda and Wolny 2013). However, the remaining *Lolium* taxa (including both geographical races of *L.multiflorum*) have synteny (Fig. 2). The syntenic patterns can be differentiated into two groups. In one (allogamous) group, the two types of rDNA sequences were located proximally on either side of the centromere of the same chromosome, as represented by *L.perenne* and both geographical forms of *L.multiflorum*. In the other (largely autogamous) group, represented by *L.persicum*, *L.temulentum*, *L.remotum*, and subspecies and races of *L.rigidum*, both types of rDNA sequences were adjacent on the same chromosome arm, with 35S always distal to 5S. *L.canariense* shows the diploid *Micropyropsis-F.pratensis* arrangement with proximally located 5S and 35S rDNA loci on separate chromosomes as well as an additional pair of 35S loci (a *Lolium* characteristic, Fig. 2). On this basis, Inda and Wolny (2013) have suggested that *L.canariense* could be the link between the *Festuca* and *Lolium* lineages.

### ﻿rDNA locus patterns among the polyploid *Festuca* species

The data presented in Fig. 2, based on the present investigation as well as earlier reports and analyses of DNA sequences (Thomas et al. 1997; Hand et al. 2010; Inda et al. 2014; Minaya et al. 2015; Ezquerro-López et al. 2017), summarise the patterns among polyploid species in subgenus Schedonorus. All the species are allopolyploid (Cao et al. 2000; Hand et al. 2010; Inda et al. 2014; Minaya et al. 2015; Ezquerro-López et al. 2017) and show no changes in the basic chromosome number (*x* = 7) and no apparent changes in the ancestral karyotype.

The numbers of 5S loci range from two in the tetraploids, *F.mairei* St. Yves, 1922 and *F.glaucescens* to eight in decaploid *F.letourneuxiana* (Festucaarundinaceavar.letourneuxiana (St. Yves) Torrecilla et Catalán, 2002) while 35S numbers ranged from one in tetraploid *F.simensis* to six in *F.letourneuxiana* (Fig. 2). Localisation of two 35S loci on the same chromosome, as in the tetraploids *F.mairei* and *F.glaucescens* (Thomas et al. 1997) is not frequently encountered in plants.

Seven of the eight *Festuca* polyploids had the 5S rDNA loci in the proximal region, either exclusively or in addition to other regions (Fig. 2). Terminal 5S loci were encountered in only three polyploid species and an interstitial 5S locus was found only in *F.simensis* (present study). In contrast, terminal 35S loci were more frequent. Five species mapped at least one 35S locus in the terminal region while four displayed exclusively terminal 35S loci (Fig. 2). Among these were tetraploids either with terminal 35S loci on each arm of one chromosome (*F.glaucescens*) or two 35S loci adjacent to each other on the same arm (*F.mairei*) (Fig. 2; Thomas et al. 1997). Three polyploids displayed exclusively proximal 35S hybridization signals including tetraploid *F.simensis* with only one 35S locus. The higher frequency of terminal 35S loci among the *Festuca* polyploids aligns well with the majority of angiosperms (Roa and Guerra 2012; Garcia et al. 2017). None of the *Festuca* species in the *Schedonorus*-*Lolium* complex studied so far have a syntenic arrangement of 5S and 35S rDNA loci, except for hexaploid *F.corsica* Salm-Reifferscheid-Dyck, 1840 which displayed synteny only in heteromorphic form (Ezquerro-López et al. 2017).

Two allotetraploids, *F.mairei* and *F.glaucescens* have been suggested as the ancestral parents of allo-octoploid *F.atlantigena* (Festucaarundinaceasubsp.atlantigena (St. Yves) Auquier, 1976) based on the formation of fertile interspecific hybrids between the two suggested ancestral parental species (Chandrasekharan and Thomas 1971) and FISH mapping of the two marker loci (Ezquerro-López et al. 2017). Six proximal 5S loci in the octoploid would reflect locus additivity from the ancestral parents while the elimination of one 35S locus may reflect genomic diploidisation. The ancestral parents of decaploid *F.letourneuxiana* could not be narrowed down by FISH mapping (Ezquerro-López et al. 2017). The allohexaploid species continental *F.arundinacea* Schreber, 1771 and *F.corsica* are hypothesised to share the same ancestral parents, *viz.*, diploid *F.pratensis* and allotetraploid *F.glaucescens* (Humphreys et al. 1995; Thomas et al. 1997; Ezquerro-López et al. 2017; Fig. 2). Two distribution patterns of 5S and 35S rDNA sequences were observed in these allohexaploids, with differential losses of 35S loci and transpositions of both 5S and 35S loci. The display of two different trajectories of speciation in allopolyploids sharing the same lower-ploid ancestors has been proposed in other angiosperms (Bao et al. 2010; Weiss-Schneeweiss et al. 2012).

All four *Festuca* higher polyploids with putative parents reveal additivity of numbers of 5S loci, but, in three cases, losses of 35S loci, (Fig. 2). Diploidisation of polyploids may lead to the evolutionary loss of repetitive sequences and duplicate copies of genes (Renny-Byfield et al. 2013). Older polyploids often, but not always, show losses of copies of 35S rDNA genes and, in allotetraploids, uniparental losses of 35S loci are common (Leitch et al. 2008; Pellicer et al. 2010; Roa and Guerra 2012; Weiss-Schneeweiss et al. 2013; Garcia et al. 2017). Although there were positional shifts involving both 5S and 35S types, the results were consistent with the general observation for angiosperms that 5S loci are less variable than 35S loci (Lan and Albert 2011; Garcia et al. 2017).

The three allotetraploids (*F.simensis*, *F.mairei* and *F.glaucescens*), as the putative sub-genome donors to the allohexaploid and octoploid species, provide a novel example of sequential allopolyploidisation. The putative progenitors of all three allotetraploids remain unknown. However, nuclear and chloroplast DNA sequence analyses (Hand et al. 2010), supported by FISH mapping (Thomas et al. 1997) indicate that a diploid sub-genome is shared between *F.mairei* and *F.glaucescens*. The tetraploid species that became the sub-genome donors for higher ploidy fescues had terminal 5S and 35S loci that were largely conserved in the derivative species (Fig. 2). Among the *Schedonorus*-*Lolium* complex diploids studied so far, none have shown terminal localization of either marker, and neither were their DNA sequences consistent with them having been progenitors of these tetraploids (Hand et al. 2010). Harper et al. (2004) speculated on the basis of molecular cytogenetic findings, that diploid *F.scariosa* Lagasca y Segura ex Willkomm, 1861, belonging to the sub-genus *Scariosae* outside the *Schedonorus*-*Lolium* complex, was a potential ancestral parent for allotetraploid *F.mairei*. The likelihood of involvement of diploid sub-genome donor species from outside the *Schedonorus*-*Lolium* complex should be further explored using molecular and cytogenetic methods, including genomic *in situ* hybridization.

The variations in numbers of 35S sites in *Lolium* and the post-polyploidisation changes in the *Festuca* species have apparently occurred without any obvious changes in the symmetrical bi-armed karyotype that is a consistent feature of the *Schedonorus*-*Lolium* complex. Such lability in the absence of obvious structural changes might be attributable to paracentric chromosome rearrangements and/or the activity of transposable elements (Datson and Murray 2006; Raskina et al. 2008; Lan and Albert 2011; Barros e Silva et al. 2013; Weiss-Schneeweiss et al. 2013; Kolano et al. 2015).

## ﻿Conclusion

This report has extended the distributional data on the rDNA sequences to seven of the ten known *Lolium* species and has added *F.simensis* to the list of seven polyploid fescue species already characterised. It has also explored the distribution patterns of rDNA loci within the *Schedonorus*-*Lolium* complex and considers some possible evolutionary trends. While these patterns can be used to deduce relationships among the higher polyploid *Festuca* species, the diploid progenitors of the allotetraploid species remain unidentified and enigmatic.

## ﻿Author contributions

HAA designed the study with AVS and WMW. HAA performed the experiment, analysed the data and wrote the manuscript with co-writing from WMW. NWE isolated the DNA and labelled all the probes for FISH. AVS and NWE provided significant help in improving the manuscript. All authors read and approved the final manuscript.
